# Shape-IT: new rapid and accurate algorithm for haplotype inference

**DOI:** 10.1186/1471-2105-9-540

**Published:** 2008-12-16

**Authors:** Olivier Delaneau, Cédric Coulonges, Jean-François Zagury

**Affiliations:** 1 Chaire de Bioinformatique, Conservatoire National des Arts et Métiers, 292 rue Saint-Martin, 75003 Paris, France

## Abstract

**Background:**

We have developed a new computational algorithm, Shape-IT, to infer haplotypes under the genetic model of coalescence with recombination developed by Stephens et al in Phase v2.1. It runs much faster than Phase v2.1 while exhibiting the same accuracy. The major algorithmic improvements rely on the use of binary trees to represent the sets of candidate haplotypes for each individual. These binary tree representations: (1) speed up the computations of posterior probabilities of the haplotypes by avoiding the redundant operations made in Phase v2.1, and (2) overcome the exponential aspect of the haplotypes inference problem by the smart exploration of the most plausible pathways (ie. haplotypes) in the binary trees.

**Results:**

Our results show that Shape-IT is several orders of magnitude faster than Phase v2.1 while being as accurate. For instance, Shape-IT runs 50 times faster than Phase v2.1 to compute the haplotypes of 200 subjects on 6,000 segments of 50 SNPs extracted from a standard Illumina 300 K chip (13 days instead of 630 days). We also compared Shape-IT with other widely used software, Gerbil, PL-EM, Fastphase, 2SNP, and Ishape in various tests: Shape-IT and Phase v2.1 were the most accurate in all cases, followed by Ishape and Fastphase. As a matter of speed, Shape-IT was faster than Ishape and Fastphase for datasets smaller than 100 SNPs, but Fastphase became faster -but still less accurate- to infer haplotypes on larger SNP datasets.

**Conclusion:**

Shape-IT deserves to be extensively used for regular haplotype inference but also in the context of the new high-throughput genotyping chips since it permits to fit the genetic model of Phase v2.1 on large datasets. This new algorithm based on tree representations could be used in other HMM-based haplotype inference software and may apply more largely to other fields using HMM.

## Background

The recent advent of genotyping chips, which can analyze up to 500,000 single nucleotide polymorphisms (SNP) per individual, offers a powerful tool for large scale association studies in human diseases. The most common approach to find genes possibly implicated in a disease relies on the comparison, in patients and controls, of the distributions of SNP markers. An approach to increase the power of such studies is to focus on more complex markers which capture implicitly the linkage disequilibrium (LD) between SNPs: the combination of SNP alleles on the same chromosome called haplotypes. Haplotypes are of great interest to study complex diseases since they are generally derived from chromosomal fragments which are transmitted from one generation to the next or which may have a biological meaning such as the promoter or the exons of a gene [1]. Beyond the biomedical applications, the comparison of haplotype distributions between populations also provides new insights in the diversity, the history and the migrations of human populations. For instance, several studies [2-6] have recently highlighted that genetic diversity of the human genome is organized in regions called haplotype blocks in which SNPs exhibit a high degree of LD and few common haplotypes. These haplotype blocks are delimited by recombination hotspots and chromosomes can thus be viewed as mosaics of common haplotypes. The recently developed HapMap project, dedicated to establish a dense map of SNPs and LD in various human populations [7-9], has emphasized the interest of haplotypes to study human diversity.

Regular genotyping (based on PCR/sequencing or on chips) provides the genotype for each SNP but does not allow the determination of the haplotypes (i.e. the combination of SNP alleles on each chromosome), and current experimental solutions to this problem are still expensive and time-consuming [10,11]. Clark was first to introduce a computational alternative [12]: the determination of haplotypes via a parsimony criterion which leads to a minimal set of haplotypes sufficient to explain the entire population. Since then, efficient statistical algorithms have been developed under the random mating assumption where the observed genotypes are formed by sampling independently two unknown haplotypes. This assumption, coupled with a probabilistic model for the haplotypes, permits to define the likelihood of the observed genotypes as a function of the model parameters. Thus, in order to infer haplotypes, the most likely parameter values are estimated via an Expectation Maximization algorithm (EM) or a Gibbs sampler algorithm (GS) on the observed genotypes.

The first EM-based model estimated the most likely haplotypes frequencies for observed genotypes without making any assumption on the mutation and recombination history of haplotypes [13]. Many software were built on this simple model and the best-known is certainly PLEM [14]. Later on, two new models were developed based on the idea that the haplotypes were arising through mutation and recombination events from few founder haplotypes. In Gerbil [15], haplotype blocks are strictly defined by dynamic programming and in each block, the haplotypes are derived through mutations from founder haplotypes. On the other hand, in Fastphase [16], in HIT [17], and in HINT [18], both mutation and recombination events on founder haplotypes are simultaneously modeled through a hidden Markov model (HMM). All these methods estimate founder haplotypes from observed genotypes via EM algorithms.

For the GS-based algorithms, the general case relies on sampling haplotypes for a genotype in function of all the haplotypes currently assigned to the other genotypes. The model of Haplotyper [19] simply favors haplotypes which have been already assigned to many genotypes. In Phase v1.0 [20], the idea was to favor the sampling of haplotypes which likely coalesce with the already assigned ones. At last, in Phase v2.1 [21,22], the sampled haplotypes are mosaics of the previously sampled ones modeled in a HMM.

Recently, an alternative approach to the statistical algorithms was proposed in 2snp [23] which computes LD measures for all pairs of SNPs and then resolves genotypes by finding the maximum spanning trees.

Several studies have suggested that the HMM-based methods were the most accurate to infer the haplotypes [17,18,24], certainly because of the flexible definition of the haplotype blocks which depends generally on the physical distance between SNPs [16]. Among the HMM-based methods, Phase v2.1 is often considered as the most accurate developed so far [24-30] which explains why it is widely used in genetic association studies [31-33] and why it was used to phase the genotype data of the HapMap project [8]. The strength of Phase v2.1 probably comes from two particularities. First, the HMM is built during the GS iterations with a number of haplotypes proportional to the number of genotypes in opposition to other HMM-based methods which define a fixed number of founder haplotypes. Second, the haplotypes are inferred by summing over all the possible hidden state sequences of the HMM (Forward algorithm) whereas many other HMM-based methods infer haplotypes by sampling only the most probable hidden sequence in the HMM (Viterbi algorithm).

However, the required running time increases dramatically with the number of SNPs since the search space grows exponentially. This prevents the easy use of Phase v2.1 in the current high-throughput chips. This fact has previously motivated us to develop Ishape [27] which matches Phase v2.1 accuracy while maintaining feasible running times. For that, we have used a two-step strategy: 1. we defined a limited space of possible haplotypes with a rapid pre-processing algorithm based on bootstrapped EM haplotypes estimations 2. on this limited set of haplotypes, we then used an accurate Phase-like algorithm. The rapidity of the first step is made possible thanks to an iterative implementation of the EM algorithm which avoids any exponential growth of the space of possible haplotypes and includes the SNPs one after the other during the computations. In practice, Ishape runs up to 15 times faster than Phase 2.1 (for up to 100 SNPs) with a similar accuracy in populations with high LD, such as Caucasian genomes.

In this work, we present major improvements which greatly reduce the computational time of Phase v2.1. These improvements have been implemented in the software package Shape-IT and compared to the widely used competitor software.

## Algorithm

### Notations (Figure 1)

**Figure 1:**
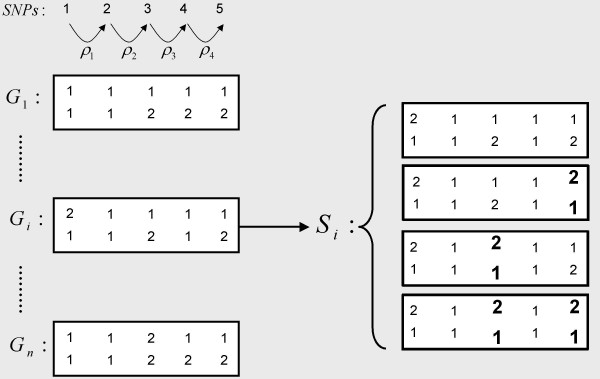
**Schematic representation of a sample of *n *genotypes**. In this example, the space of possible haplotypes *S*_*i *_for individual *i *contains 4 haplotype pairs with 8 distinct haplotypes. The possible phases between heterozygous markers are shown in bold.

Let's assume we have a sample of *n *genotypes *G *= {*G*_1_,..., *G*_*n*_} describing the allelic content of *n *diploid individuals over *s *SNPs. A genotype is split into a haplotype pair by setting the phases between the *z *heterozygous SNPs (*z *≤ *s*). The number of distinct haplotype pairs consistent with a genotype is then 2^(*z*-1)^. Let *S *= {*S*_1_,..., *S*_*n*_} denotes the total haplotype space where *S*_*i *_is the space of possible haplotype pairs associated with the *ith *genotype. Moreover, let's assume we have the recombination parameters *ρ *= {*ρ*_1_,..., *ρ*_*s*-1_} in the *s-1 *intervals between the *s *SNPs of the sample as described by Stephens et al [22].

### Gibbs sampler algorithm

The GS algorithm considers the haplotype reconstructions of *n *individuals as a set of *n *random variables *H *= {*H*_1_,..., *H*_*n*_} with sampling spaces in *S *and it estimates the conditional joint distribution of *H *given *G *and some recombination parameters *ρ*: Pr(*H *| *G*, *ρ*). In simple words, it computes a conditional probability for each haplotype pair of *S *in light of the observed genotypes *G *and the recombination pattern between the SNPs. Given these probabilities, the haplotype frequencies and the most likely haplotype pair for each genotype are straightforward to obtain. In practice, Pr(*H *| *G*, *ρ*) is estimated by sampling from the stationary distribution of a Gibbs sampler (GS) *H*^(0)^,..., *H*^(*t*)^,... where a state *H*^(*t*) ^is a particular realization of the random variables of *H*: *n *haplotype pairs from *S *which resolves the *n *genotypes of *G*. The GS starts with a random haplotype realization *H*^(0)^, and goes from *H*^(*t*) ^to *H*^(*t*+1) ^by updating the haplotype pair of an individual *i *in light of the *2n-2 *other haplotypes found in *H*^(*t*)^, that we call $H_{-i}^{\left(t\right)}$. This "haplotypes update" step is done by sampling a new haplotype pair from the conditional distribution Pr(*H*_*i *_| $H_{-i}^{\left(t\right)}$, *ρ*) proposed by Fearnhead and Donnelly [34] and Li and Stephens [35]. This conditional distribution, called FDLS distribution in the following, is computed thanks to a hidden Markov model for haplotypes described in the next section. The important fact here is that computation of Pr(*H*_*i *_| $H_{-i}^{\left(t\right)}$, *ρ*) constitutes the most time-consuming part of the GS since it has to be done on a space of possible haplotype pairs which grows exponentially with the number of heterozygous SNPs.

An iteration of the GS algorithm corresponds to update successively the haplotypes of the *n *individuals of *G *given a randomly initialized order of treatment. Between iterations, according to the Metropolis Hasting acceptance rates described by Stephens et al [22], we accept or reject (1) new values for the recombination parameters *ρ *= {*ρ*_1_,..., *ρ*_*s*-1_} in the *s-1 *intervals between SNPs and (2) new treatment order of genotypes in the GS. To finally obtain Pr(*H *| *G*, *ρ*), we discard the first iterations of the GS as burn-in iterations (typically 100) and for the *n *genotypes *G*_*i*_, we average the distribution Pr(*H*_*i *_| $H_{-i}^{\left(t\right)}$, *ρ*) on several main iterations (typically 100).

### Computation of a haplotype pair probability in a HMM (Figure 2)

**Figure 2:**
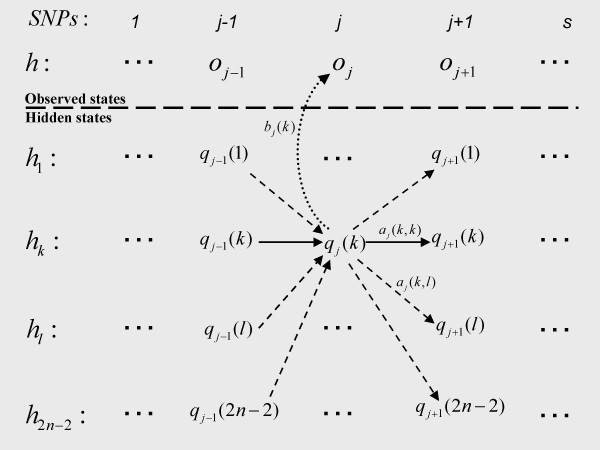
**Representation of the execution trellis of the hidden Markov model used to compute the probability of a haplotype**. The haplotypes *h*_1_,..., *h*_2*n*-2 _denote the previously sampled haplotypes which are used to compute the probability of the observed haplotype *h*. The sets {*o*_1_,..., *o*_*s*_} and {*q*_1_(*k*), ..., *q*_*s*_(*k*)} correspond respectively to the observed state sequence of haplotype *h *and to the hidden state sequence of haplotype *h*_*k*_. The transition probability *a*_*j*_(*k*,*l*) corresponds to the probability of jumping from hidden state *q*_*j*_(*k*) of haplotype *h*_*k *_to hidden state *q*_*j*+1_(*l*) of haplotype *h*_*l*_, and the emission probability *b*_*j*_(*k*) corresponds to the probability of observing *o*_*j *_given the hidden state *q*_*j*_(*k*). To compute the probability of observing the sequence h = {*o*_1_, ..., *o*_*s*_} in this HMM, one must sum up the probabilities of observing *h *over all (2*n *- 2)^*s *^possible sequences of *s *hidden states which is done efficiently by the forward algorithm.

First of all, we assume that genotypes are produced by sampling independently two haplotypes according to their respective probabilities, which yields:

(1) $$\mathrm{Pr}\left(H_{i}=\left(h,h^{\prime }\right)|H_{-i}^{\left(t\right)},\rho \right)=\left(2-\delta_{h,h^{\prime }}\right)\pi \left(h|h_{1},...,h_{2n-2},\rho \right)\pi \left(h^{\prime }|h_{1},...,h_{2n-2},\rho \right)$$

where *δ*_*h*,*h*' _= 0 if *h *≠ *h*' and *δ*_*h*,*h' *_= 1 if *h *= *h'*. The conditional probability *π *of haplotype *h *reflects how likely *h *corresponds to an "imperfect mosaic" of the other haplotypes {*h*_1_, ..., *h*_2*n*-2_} [22]. The underlying idea is that haplotype *h *has been probably created through the generations as a recombined sequence of haplotypes from the pool {*h*_1_, ..., *h*_2*n*-2_}, possibly altered by some mutations. One models this by computing the probability of observing the sequence *h *= {*o*_1_, ..., *o*_*s*_} in a hidden Markov model *λ *designed to represent all possible mosaics of {*h*_1_, ..., *h*_2*n*-2_}: *π*(*h*|*h*_1_, ..., *h*_2*n*-2_, *ρ*) = Pr(*o*_1_, ..., *o*_*s*_|*λ*). Such HMM *λ *can be viewed as a trellis of *s *× (2*n *- 2) hidden states *q*_*j *_(*k*) with 1 ≤ *j *≤ *s *and 1 ≤ *k *≤ 2*n*-2. A hidden state *q*_*j*_(*k*) of *λ *corresponds to the allele of haplotype *h*_*k *_at SNP *j *and it is linked to all the hidden states *q*_*j*+1_(*l*) (1 ≤ *l *≤ 2*n*-2) at SNP *j+1 *in order to model all the possible recombination jumps of haplotypes between SNPs *j *and *j+1 *(Figure 2). Then, a sequence of *s *hidden states in *λ *through the *s *SNPs corresponds to a particular mosaic of {*h*_1_, ..., *h*_2*n*-2_}. The probability of observing *h *= {*o*_1_, ..., *o*_*s*_} in *λ *is computed thanks to transition probabilities between hidden states which mimic recombination and thanks to emission probabilities from hidden alleles to observed alleles which mimic mutation. Similar hidden Markov models have been proposed, but they generally rely on a limited number of founder haplotypes where the most likely transition and emission probabilities are estimated from observed genotype data via an EM algorithm [17,18]. Here, the emission and transition probabilities are defined with prior distributions depending respectively on a constant mutation parameter and on the variable recombination parameters *ρ *. The objective of this section is not to fully describe the probabilistic model of transitions and emissions since this has already been done by Stephens and Scheet [22]. Instead, we focus on how the haplotype probability is computed in such a HMM *λ *from transition and emission probabilities. We thus assume that the following quantities are known as set up by Stephens and Scheet:

• The transition probability *a*_*j *_(*l*,*k*) from the state *q*_*j*_(*l*) of haplotype *h*_*l *_for SNP *j *to the state *q*_*j*+1_(*k*) of haplotype *h*_*k *_for SNP *j+1*. If *l *≠ *k *then *a*_*j *_(*l*,*k*) is the probability for *h*_*l *_to be recombined with *h*_*k *_between SNP *j *and SNP *j+1 *(large dashed arrows in Figure 2). And conversely, if *l *= *k *then *a*_*j *_(*l*,*l*) is the probability for *h*_*l *_to be not recombined between the two SNPs (plain arrows in Figure 2).

• The emission probability *b*_*j*_(*k*) of the hidden allele of *q*_*j*_(*k*) in the observed allele *o*_*j *_of *h *(small dashed arrows in Figure 2). If the hidden allele is different from the observed one, then *b*_*j*_(*k*) corresponds to the probability that the hidden allele *q*_*j*_(*k*) has been altered in *o*_*j *_by a mutation event. Else, *b*_*j*_(*k*) corresponds to the probability that no mutation has occurred.

In the HMM *λ*, the probability of a hidden states' sequence is given by the product of the corresponding transition probabilities. And the probability to observe *h *= {*o*_1_, ..., *o*_*s*_} given a particular hidden states' sequence is obtained by the product of the probabilities for the hidden alleles to be emitted in the observed ones. Finally, to compute the probability Pr(*h*|*λ*), one must sum up the probabilities of observing *h *over all (2*n *- 2)^*s *^possible sequences of *s *hidden states. An alternative to this expensive computational approach is to define a forward probability *α*_*j*_(*k*) as the probability for the incomplete observed sequence {*o*_1_, ..., *o*_*j*_} to be emitted by all the possible hidden sequences that end at state *q*_*j*_(*k*). Then, the partial posterior probability *π*_*j *_until SNP *j *of *h *can be written as follows:

(1a) $$\pi_{j}(h|h_{1},...,h_{2n-2},\rho )=\sum_{k=1}^{2n-2}\alpha_{j}(k)$$

And the total probability of *h *over the *s *SNPs becomes:

(2) *π*(*h*|*h*_1_, ..., *h*_2*n*-2_, *ρ*) = *π*_*s*_(*h*|*h*_1_, ..., *h*_2*n*-2_, *ρ*)

The computations of *α*_*j*_(*k*) for *k *= 1,..., 2*n*-2 and *j *= 1,..., *s *are efficiently done by a recursive algorithm for HMM called forward algorithm [36]. It starts from initial values:

(3) *α*_1_(*k*) = *b*_1_(*k*)/(2*n *- 2)

And recursively computes the *α*_*j*+1 _values from the *α*_*j *_values as follows:

(4) $$\alpha_{j+1}(k)=b_{j+1}(k)\times \sum_{l=1}^{2n-2}\left[\alpha_{j}(l)\times a_{j}(l,k)\right]$$

Computing all the *α *values for a haplotype requires now running time in *O*(*sn*^2^) instead of *O*(*n*^*s*^).

### Computation of the FDLS distribution from a haplotype list by Phase v2.1 (Figure 3A)

**Figure 3:**
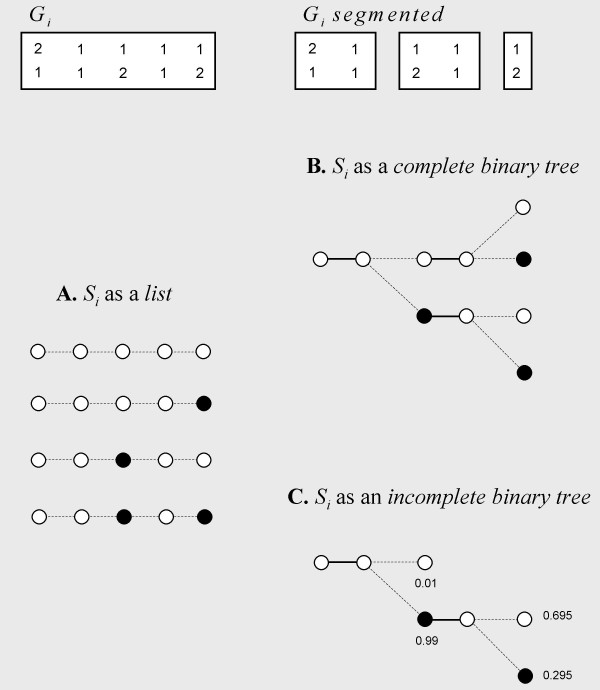
**Different representations of the space of possible haplotypes pairs S_*i*_**. The left panel (A) shows the list representation commonly used by haplotype software such as Phase v2.1. The lower right panel (C) shows the representation used by Shape-IT. White and black circles indicate the phases between the heterozygous SNPs. On this example we use the same genotype G_*i *_described in Figure 1. For iterations as performed by Phase v2.1 (A), the list requires the exploration of 20 nodes (4 haplotype pairs × 5 SNPs). With the complete tree representation (B) 10 nodes need to be explored, and with the incomplete tree representation as performed by Shape-IT (C), only 7 nodes need to be explored. The difference observed between (B) and (C) results from the pruning strategy which avoids the exploration of the nodes with probability ≤ 0.01.

The Phase v2.1 algorithm considers the haplotype space *S*_*i *_as a list of $2^{z_{i}}$ haplotypes compatibles with the genotype *G*_*i *_where *z*_*i *_is the number of heterozygous SNPs. And it computes the FDLS distribution over this list with equations (3) and (1) on the HMM *λ*. This approach is computationally intensive for two reasons. First, it performs many times the same computations of *α *values with the forward algorithm since the haplotypes of *S*_*i *_are derived from the same genotype and share thus identical allelic segments. For instance, as shown in Figure 3A, several haplotypes of *S*_*i *_differ only in the last SNPs while the computation of forward values *α *starts each time from the first SNP. Second, the list of haplotypes grows exponentially with the number of heterozygous SNPs which prevents any application with a high number of SNPs. To partially overcome this problem, a "divide for conquer" solution called "partition-ligation" (PL) was first proposed by Niu et al [14,19,21]. It has been included in the Phase v2.1 algorithm as follows: it first divides the genotypes into segments of limited size (typically 5–8 SNPs), determines the most probable haplotypes on each segment with complete runs of the GS, and then progressively ligates haplotypes of the adjacent segments in several runs until completion. When two adjacent segments are ligated, the space *S *of candidate haplotype pairs is initialized from all combinations of the most probable haplotypes previously found in each segment. However, the PL procedure remains computationally expensive because it implies *2s/p - 1 *(where *p *is the size of the partitions) complete runs of the algorithm, each time on a quadratic number of combinations of adjacent plausible haplotypes.

### Computation of the FDLS distribution from a complete binary tree by Shape-IT (Figure 3B)

To compute the FDLS distribution while avoiding any redundant calculations of *α *values, our algorithm uses a complete binary tree (called haplotype tree in the following) instead of an exhaustive list to represent the haplotype pairs space *S*_*i*_. It can be viewed as an extension of the forward algorithm which computes the probabilities of observing in the HMM *λ *several pairs of sequences classified into a binary tree rather than observing a unique sequence.

Such a haplotype tree is easily derived from a partition of genotype *G*_*i *_into *m *unambiguous segments $G_{i}=\{(g_{1},{g^{\prime }}_{1}),...,(g_{m},{g^{\prime }}_{m})\}$ : each one starts from a heterozygous SNP, includes all the following homozygous SNPs, and ends before the next heterozygous SNP. A node of the haplotype tree corresponds to a genotype segment $\left(g_{j},{g^{\prime }}_{j}\right)$, and the two children nodes, to the two possible switch orientations with the following segment (*g*_*j*+1_, ${g^{\prime }}_{j+1}$) and (${g^{\prime }}_{j+1}$, *g*_*j*+1_). Then, a single path from the root to a leaf corresponds to a single possible haplotype pair of *S*_*i *_(Figure 3B).

**Figure 4:**
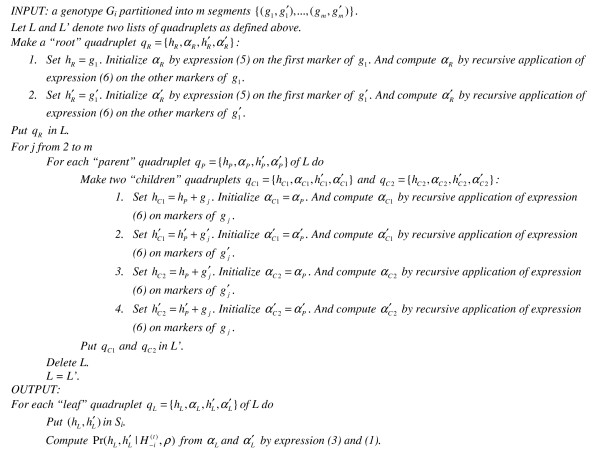
Algorithm 1 to compute the FDSL distribution on the complete haplotype tree.

To compute efficiently the FDLS distribution, Shape-IT explores the haplotype tree with a single recursive algorithm which combines the reconstruction of the haplotypes and the calculation of associated *α *forward values. In practice, it iterates the nodes by level-order (i.e. segment-order) to avoid any previous construction in memory of the haplotype tree. When visiting a node with the associated genotype segment (*g*, *g*'), the algorithm makes recursively a quadruplet *q *= {*h*, *α*, *h'*, *α'*} where *h *and *h*' are the two haplotypes with respective forward values *α *and *α' *corresponding to the current explored path in the haplotype tree. Once all the nodes visited, the haplotype pairs of *S*_*i *_and the FDLS distribution are given respectively by the haplotypes and the forward values of the quadruplets associated to the leaf nodes. This approach is implemented in the algorithm 1 (Figure 4).

This algorithm avoids all the unnecessary forward value computations made when using the representation by haplotype lists. However, the haplotype tree to be explored still grows exponentially with an increasing number of heterozygous SNPs. It results in a list *L *whose size is multiplied by two at each level explored (Figure 4). As with the classical haplotype list approach, this algorithm can be simply implemented in a PL strategy: first, a haplotype tree is derived for each segment of genotype, and then the most probable adjacent subtrees are determined and combined until completion. We have used an alternative strategy described in the next paragraph.

### Computation of the FDLS distribution from an incomplete binary tree by Shape-IT (Figure 3C)

**Figure 5:**
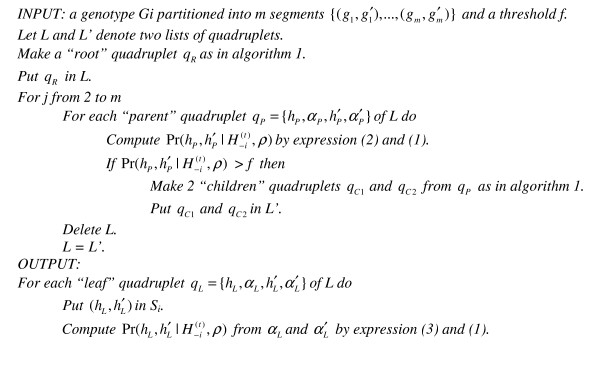
Algorithm 2 to compute the FDSL distribution on the incomplete haplotype tree.

In practice, the number of haplotype pairs sufficiently probable to be sampled in the FDLS distribution is roughly linear with the number of SNPs instead of being exponential. As an alternative to the classical and expensive PL strategy, we have thus modified our recursive algorithm to explore only the paths in the haplotype tree which correspond to the most plausible haplotype pairs. In other words, our algorithm aims at identifying an incomplete binary tree of limited size which captures at best the informative part of FDLS distribution (Figure 3C). For that, recursions are made only on nodes exhibiting a probability, as given by expressions (2) and (1), greater than a threshold *f *initially defined. In practice, it results in maintaining a list *L *of quadruplets of limited size for each level of the tree explored, which no longer grows exponentially with the number of heterozygous SNPs. The corresponding modifications made in algorithm 1 are implemented in algorithm 2 (Figure 5). Obviously the value of the threshold *f *affects the number of quadruplets kept at each level of the haplotype tree and thus, the number of haplotype pairs on which the FDLS distribution is computed. It is clear that the value of threshold *f *influences the diversity of haplotypes to be captured and so, the computational effort needed. However, the strength of our algorithm clearly lies in the greatly reduced complexity with the number of SNPs of the FDLS computation step. Moreover, compared to the *2s/p - 1 *complete runs of the GS required by the PL strategy, it treats all the SNPs in a single run.

## Methods

We have implemented our algorithm in the software package Shape-IT publicly available at . We have extensively compared Shape-IT with the widely used haplotype inference software 2snp [23], Gerbil [15], Fastphase [16], PL-EM [14], Ishape [27] and Phase v2.1 [21,22] on 3 kinds of datasets described hereafter. All the software were run with default parameters on a standard 2 GHz computer with 1 Go of RAM.

In the comparisons, we have tried to work as close as possible to real conditions: on the one hand, we have used tightly linked SNPs such as those used in a single gene fine mapping and on the other hand, we have used TagSNPs with a low level of LD which correspond to the worst conditions to infer haplotypes. At last, we have also made estimations of the running times required by the most accurate software to infer the haplotypes of a 300 K Illumina chips.

### Single gene datasets

First, we have used genotypes for which the haplotypes have been completely determined experimentally: the GH1 [37] and ApoE [38] genes. The GH1 dataset contains 14 SNPs for 150 Caucasian individuals and the ApoE dataset contains 9 SNPs for 90 individuals of mixed ethnic origins. For each gene, we have additionally generated 100 replicates by randomly masking 5% of the alleles in order to simulate real experimental conditions (missing data). On these datasets, we have measured the IER (Individual Error Rate) and the MER (Missing data Error Rate) which corresponds respectively to the percentage of individuals incorrectly inferred and to the percentage of missing data incorrectly inferred. Although of limited size, these two genes are very useful to compare precisely the haplotype frequency estimations made by the algorithms via the I_F _coefficient [25], since haplotype frequencies are commonly used by the geneticists in genetic association studies.

### HapMap trio datasets

Second, we have worked on trios' genotypes (2 parents and 1 child) derived from the HapMap project [7,8]. We have collected five regions of 10 Mb on chromosomes 1, 2, 3, 4 and 5 in African (YRI) or European (CEU) populations. The 10 resulting chromosomal regions have been preprocessed by the Haploview software [39] to remove SNPs with Mendelian inconsistency or with insufficient minor allele frequency (MAF). From these chromosomal regions, we have generated several HapMap datasets according to the choices of markers described in Table 1[24,27]. On all these trios' genotypes, the parent haplotypes can be partially obtained (about ~80% of the phases between adjacent heterozygous SNPs are determined), and we have measured the running times of the various algorithms and the SER (Switch Error Rate) of haplotypes inferred by the various software. The SER corresponds to the percentage of known phases between adjacent heterozygous SNPs (obtained thanks to the trios affiliation) incorrectly inferred [22,27], which is more adapted than the IER on large numbers of SNPs because the IER does not differentiate between one or several heterozygous SNPs incorrectly inferred.

**Table 1:** Hapmap trio datasets description

**Datasets**	**Chromosome**	**#datasets**	**#SNP**	**#indiv**	**Details**
CEU Size	1 to 5	250	10 to 160	60	50 datasets of 10, 20, 40, 80 and 160 adjacent SNPs with MAF above 5%
CEU Density	1 to 5	300	40	60	50 datasets with spanned distance between SNP above 0, 0.5, 1, 2, 4 and 8 kb (MAF 5%)
CEU MAF	1 to 5	150	40	60	50 datasets with MAF above 1%, 5% and 10%
YRI Size	1 to 5	250	10 to 160	60	50 datasets of 10, 20, 40, 80 and 160 adjacent SNPs with MAF above 5%
YRI Density	1 to 5	300	40	60	50 datasets with spanned distance between SNP above 0, 0.5, 1, 2, 4 and 8 kb (MAF 5%)
YRI MAF	1 to 5	150	40	60	50 datasets with MAF above 1%, 5% and 10%
CEU illumina 50	12	300	50	60	15,000 illumina SNPs grouped by dataset of 50 SNPs
CEU illumina 100	12	150	100	60	15,000 illumina SNPs grouped by dataset of 100 SNPs
CEU illumina 200	12	75	200	60	15,000 illumina SNPs grouped by dataset of 200 SNPs
GRIV	1	90	50 to 200	100 to 300	3,500 illumina SNPs grouped by dataset of 50, 100 and 200 SNPs

Description of the benchmarks derived from the HapMap trios datasets that we used to compare accuracy and runtimes of the various algorithms in Table 4. For each parameter (size, density, and MAF) 10 samples were chosen in each of the chromosomes 1 to 5, i.e. a total of 50 tests per parameter.

To investigate on the impact of low LD in haplotype inference, we have also used a set of 15,000 adjacent Tag SNPs picked up from the large arm of chromosome 12 and found in the 300 K Illumina chips.

### GRIV cohort datasets

Third, we have generated large SNP datasets from subjects of the GRIV (Genomics of Resistance to Immunodeficiency Virus) cohort genotyped with the 300 K Illumina chip. The GRIV cohort comprehends about 400 Caucasian subjects collected for genomic studies in AIDS [1,40-43]. These datasets were used to estimate the running times required by the most accurate software to infer the haplotypes of a 300 K Illumina chips. For that, we have generated 10 datasets from the GRIV cohort data for various numbers of markers (50, 100 and 200) and for various numbers of individuals (100, 200 and 300). Then the average running time over the 10 datasets of each combination of SNP number and genotype number was used to extrapolate the running time required to infer the haplotypes over the 300,000 SNPs.

## Results

### Empirical determination of the threshold f (Figure 6)

**Figure 6:**
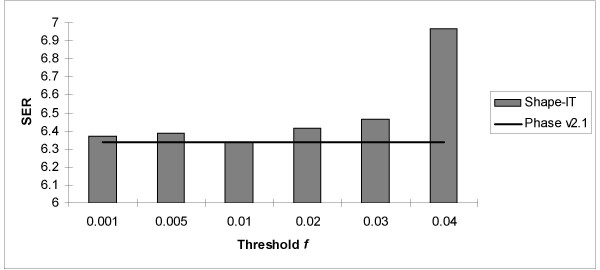
**Accuracy of the different values tested for the threshold *f *in Shape-IT (grey boxes) compared to Phase v2.1 (black line)**. This comparison was done on 300 datasets of 50 Tag SNPs called CEU Illumina 50.

As discussed in the section Algorithm, Shape-IT relies on a threshold *f *to discard some branches of the haplotype binary trees. So, we have tested several values for *f*: the accuracy is clearly stable for values below 0.01. Since the running time was optimal for *f *= 0.01, we have used this value as default in all the following comparisons.

### Comparisons on the single gene datasets (Table 2 and 3)

**Table 2:** Results obtained by various haplotyping software on the experimentally determined ApoE dataset.

**ApoE**	**0%MD**		**5%MD**		
	**IER**	**IF**	**IER**	**MER**	**IF**
		
2snp	20.0	83.8	22.7	7.3	83.9
Fastphase	11.3	89.4	17.4	6.1	87.5
Gerbil	20.0	81.3	20.3	6.6	84.6
Ishape	***5.6***	***94.1***	***10.2***	5.9	***92.5***
Shape-IT	***5.6***	***94.1***	10.5	6.2	92.4
Phase v2.1	5.8	94.0	***10.2***	***5.8***	92.4
PLEM	12.5	89.8	16.0	6.5	88.7

For the various software tested, we measured the percentage of individuals incorrectly reconstructed (IER), the percentage of missing data incorrectly inferred (MER), and the distance between real and inferred haplotype frequencies (IF) on the ApoE with complete genotypes and 5% random missing genotypes.

**Table 3:** Results obtained by various haplotyping software on the experimentally determined GH1 dataset.

**GH1**	**0%MD**		**5%MD**		
	**IER**	**IF**	**IER**	**MER**	**IF**
		
2snp	15.7	88.2	22.0	7.5	88.3
Fastphase	10.5	92.5	17.3	4.5	90.7
Gerbil	11.8	92.8	16.7	***4.2***	91.6
Ishape	***10.1***	***93.8***	15.0	4.5	***92.6***
Shape-IT	10.3	93.6	***14.9***	4.5	92.5
Phase v2.1	10.3	93.7	15.2	4.5	92.5
PLEM	12.4	90.3	17.2	4.8	89.4

For the various software tested, we measured the percentage of individuals incorrectly reconstructed (IER), the percentage of missing data incorrectly inferred (MER), and the distance between real and inferred haplotype frequencies (IF) on the GH1 with complete genotypes and 5% random missing genotypes.

On these datasets, Shape-IT, Ishape and Phase v2.1 give clearly the better haplotype reconstructions and frequency estimations compared to the other software. One can notice that Ishape seems to be slightly more accurate than Shape-IT and Phase v2.1. For the completion of missing data, all the methods (except 2snp) are closely related.

### Comparisons on the HapMap trio datasets (Table 1 and 4)

**Table 4:** Hapmap trio datasets results

**Datasets**	**Shape-IT**		**Phase v2.1**		**Fastphase**		**Ishape**		**2snp**		**Gerbil**		**PLEM**	
							
	**SER**	**Time**	**SER**	**Time**	**SER**	**Time**	**SER**	**Time**	**SER**	**Time**	**SER**	**Time**	**SER**	**Time**
CEU Size	***1.1***		***1.1***		1.5		**1.1**		2.2		2.3		2.0	
		53		832		113		93		< 1		50		10
YRI Size	***1.7***		***1.7***		2.3		1.8		4.5		3.9		4.2	
		64		1,209		125		138		< 1		131		10
CEU Density	***2.3***		***2.3***		2.7		2.4		4.2		4.0		4.1	
		26		214		64		43		< 1		5		6
YRI Density	***3.7***		***3.7***		4.9		3.9		8.5		7.5		8.8	
		35		490		71		80		< 1		9		5
CEU MAF	***1.1***		***1.1***		1.2		1.2		2.0		2.1		1.7	
		19		104		71		22		< 1		2		4
YRI MAF	***1.5***		***1.5***		2.0		***1.5***		4.5		3.8		3.2	
		26		173		80		38		< 1		4		4
CEU 50 illumina SNP	***6.3***		***6.3***		7.2		6.6		10.7		9.2		12.2	
		51		1,214		60		161		< 1		22		5
CEU 100 illumina SNP	***6.7***		6.8		7.7		9.2		11.3		9.7		N/A	
		143		11,678		144		461		< 1		254		N/A
CEU 200 illumina SNP	***7.2***		N/A		8.0		N/A		11.5		9.9		N/A	
		372		N/A		198		N/A		< 1		2,038		N/A

N/A: software was unable to handle some of these datasets (errors or untracktable running times). Results of the various tested software on the HapMap trios datasets described in Table 1. For each software tested, the mean percentage of heterozygous markers incorrectly inferred (SER) is shown in the upper-left corner, and the mean running time in seconds is shown in the lower-right corner.

As a matter of accuracy, Shape-IT and Phase v2.1 outperform all the other methods. Ishape comes second but plunges when dealing with larger number of Tag SNPs. Fastphase comes third but it seems to work relatively better when the datasets get bigger. 2snp, Gerbil, and PLEM do not match the accuracy of the other software. All the software get higher error rates when the number of Tag SNPs increases which is probably the consequence of the increasing complexity of the LD pattern when dealing with limited numbers of individuals.

As a matter of speed, the fastest software is clearly 2snp. For relatively small numbers of SNPs, PLEM and Gerbil are also very fast, but become very slow when the number of SNPs increases or when the LD pattern gets more complex to capture. Among the 4 most accurate software (Phase v2.1, Fastphase, Ishape, and Shape-IT), Phase v2.1 is the slowest, Shape-IT is the fastest for small and medium-sized SNP samples (< 100 SNPs), and Fastphase becomes faster for larger numbers of SNPs (see additional file 1).

### Running time on the GRIV cohort datasets (Table 5)

**Table 5:** Comparison of the estimated running times of various software on 300 K Illunima genotyping chips datasets.

**#SNPs**	**#genotypes**	**Fastphase**	**Ishape**	**Shape-IT**	**Phase v2.1**
50	100	10	29	10	151
100	100	6	37	12	519
200	100	6	41	19	3,137
50	200	21	34	13	443
100	200	21	119	29	2,739
200	200	21	124	37	7,601
50	300	37	113	28	1,372
100	300	41	268	52	6,514
200	300	42	261	81	12,757

Estimations of the running times in days of the 4 most accurate software (Phase v2.1, Ishape, Fastphase and Shape-IT) to infer the haplotypes for 100, 200, or 300 genotypes derived from Illumina 300 k chips partitioned into segments of either 50 SNPs, or 100 SNPs, or 200 SNPs. For each combination #SNPs #genotypes, the running time estimations were extrapolated from the measures performed on 10 datasets extracted from the GRIV cohort 300 K Illumina chip genomic data.

On these datasets, Shape-IT runs between 15 to 150 times faster that Phase v2.1, depending on the segmentation strategy used (50, 100 or 200 SNPs) and the number of genotypes in the population (100, 200 or 300). Fastphase remains the fastest software but closely followed by Shape-IT. The increase of SNP and genotype numbers strongly cripples Phase v2.1 and Ishape, while it is better handled by Shape-IT and Fastphase.

## Discussion and conclusion

We have developed a new algorithm derived from the Phase v2.1 Gibbs sampler scheme. We have improved the most time-consuming steps by using binary tree representations and by avoiding the PL procedure thanks to an incomplete exploration of binary trees. The resulting software, Shape-IT, is extremely accurate like Phase v2.1, but may run up to 150 times faster as shown in our tests. These results have an impact for the computation of haplotypes in genome scans as shown in Table 5. As an example, for the 300,000 SNPs of an Illumina genotyping chip, inferring haplotypes on 6,000 segments of 50 SNPs with a regular 2 GHz computer would take for Shape-IT about 10 days for 100 individuals, 13 days for 200 individuals, 28 days for 300 individuals while it would take for Phase v2.1 151 days for 100 individuals (15 times more), 443 days for 200 individuals (34 times more) and 1372 days for 300 individuals (49 times more). The gain of time using Shape-IT is thus considerable and practically very useful to exploit datasets derived from large-scale genotyping chips.

An important aspect of this work is that other haplotype inference software relying on HMM may gain to implement this new binary tree representation of the observed genotypes. Moreover, we have not found in the literature the description of this algorithm whereas it might be useful for other fields using HMM.

## Availability and requirements

Project name: Shape-IT v1.0

Project home page: 

Operating systems: MacOS, Windows, Linux32bits and Linux64bits.

Programming language: C++

Do not forget to read the manual file, manual_ShapeITv1.0.pdf, to get the detailed information. The software remains confidential until publication of the work. It will be freely available to academics, and a licence will be needed for non-academics (patented for business and commercial applications).

## Authors' contributions

OD and CC worked on developing the methods and programs used in this study under the direct supervision of JFZ who conceived the study. All the authors have read and approved the final manuscript.

## Supplementary Material

Additional file 1 **Detailed trio datasets results.** Detailed results of the various software tested on the HapMap trios datasets described in Table 1. For each software tested, the mean percentage of heterozygous markers incorrectly inferred (SER) and the average running time in seconds are shown. Click here for file
